# Unexpected diagnosis of basal cell carcinoma in a patient presenting with a secondary location of *Leishmania* parasites in the skin

**DOI:** 10.11604/pamj.2019.34.8.18946

**Published:** 2019-09-03

**Authors:** Imène Ben Abda, Rym Ben-Abdallah, Houda Hammami, Meriem Ben Abid, Hela Mdimagh-Kchir, Karim Bahri, Emna Siala, Karim Aoun, Aida Bouratbine

**Affiliations:** 1 Department of Parasitology, Pasteur Institute of Tunis, Tunisia; 2 Research Laboratory Medical Parasitology, Biotechnology and Biomolecule LR11IPT-06, University Tunis El-Manar, Pasteur Institute of Tunis, Tunisia; 3 Department of Dermatology, Habib Thameur Hospital, Tunis, Tunisia; 4 Private Laboratory of Pathology, Tunis, Tunisia; 5 Private Medical office, Infectious Diseases, Tunis, Tunisia

**Keywords:** Basal cell carcinoma, leishmaniasis, *Leishmania infantum*, real time PCR, skin leishmaniasis

## Abstract

We report here a case of simultaneous cutaneous and visceral manifestations due to *Leishmania L. infantum* diagnosed in an immunocompetent adult. We describe a 74-year-old woman from Tunis, Tunisia, who presented a biologically confirmed visceral leishmaniasis infection concomitant with arm ulceration which appeared 2 years before. *Leishmania* DNA was detected by ITS PCR in both buffy coat and dermal scrapping of the arm lesion. Sequencing revealed that the 2 isolated strains corresponded to *L. infantum* and were 100% identical. The symptoms of visceral leishmaniasis responded to amphotericin B with rapid healing. However, the skin lesion did not improve although *Leishmania* PCR on dermal sample became negative. This location is probably secondarily to lymphatic or blood dissemination during the systemic visceral leishmaniasis infection. It would be favored by the inflammatory environment induced by the basal cell carcinoma subsequently diagnosed.

## Introduction

In Tunisia, two clinical forms of Leishmaniasis are endemic [1,2]. Cutaneous leishmaniasis (CL) results in skin lesions at the site of the infective sandfly bite. Visceral leishmaniasis (VL) is a more serious systemic infection associated with high morbidity and not negligible mortality rates. It is characterized by prolonged fever, splenomegaly, pancytopenia and hypergammaglobulinemia [1,3]. Three *Leishmania* species are responsible of CL cases in Tunisia [2]. *Leishmania (L.) major*, largely distributed in the centre and the south of country, is the most prevalent with thousands of cases per year [2]. *Leishmania infantum* and L. tropica are less prevalent and limited mainly to the Northern and the southeastern regions respectively with an incidence of 50-150 cases per year for each [2]. *Leishmania infantum* is also the single etiological agent of VL in Tunisia. The annual VL incidence does not exceed 50 to 100 cases mainly reported in children under five-years old originating from the northern and central parts of the country [3]. Nevertheless, adult cases, immuno-compromised and even immuno-competent, have been increasingly reported over the last years with a proportion of around 10% of all VL cases [3]. Visceral leishmaniasis due to *L. infantum* is not generally associated with cutaneous ulcers [1,2]. The occurrence, concomitantly or not, of both CL and VL due to *L. infantum* is a rarity described in some immuno-compromised patients, notably HIV co-infected [4]. The current report is the first case of a Tunisian immunocompetent adult with both cutaneous and visceral manifestations of leishmaniasis.

## Patient and observation

**Figure 1:**
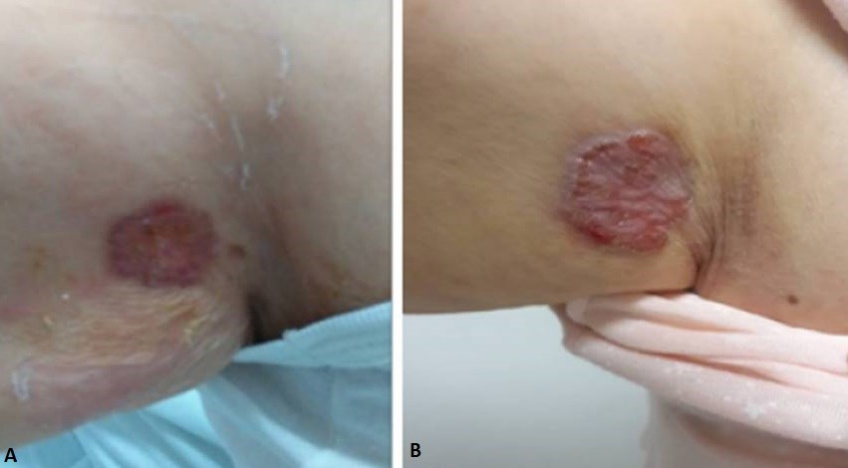
Lesion aspect before (A) and after *leishmania* specific treatment (B)

A 74-year-old woman, living in Tunis, Tunisia, was admitted for an investigation of fever evolving since weeks accompanied by sweating and chills. Oral temperature ranging from 38 to 39°C had been regularly measured. She also complained of fatigue, diarrhea, unclear abdominal pain and occasional non-productive cough. Her sole medical history was a well-controlled hypertension. Physical examination was poor, revealing a palpable spleen just below the left costal margin after deep inspiration. A well-circumscribed, erythematous plaque with a thin rolled border and large erosions and crusts on the surface A single well-defined, ulcer surrounded by erythematous edging, with serous oozing (3x3cm in size), was observed at her right arm near the axilla (Figure 1 A). The patient reported that the lesion was painless without tendency to heal, but could not precisely recall when it had first appeared (2 or 3 years). The rest of the physical examination was essentially normal. Otherwise, the patient presented neither risk factors of HIV infection nor chronic disease, transfusion or immunosuppressive therapy. Laboratory findings on admission demonstrated in the blood count a pancytopenia with normocytic normochromic anemia (hemoglobin of 8.7 g/dL), leucocytopenia (2900/mm^3^) and thrombocytopenia (113000/mm^3^). Sedimentation rate was 115mm (1^st^ hour), C-reactive protein 215mg/l and procalcitonine <0.5 ng/ml. Serum Protein Electrophoresis displayed an abnormal pattern with a hypoalbuminemia at 31.1 g/l and a monoclonal hypergammaglobulinemia at 38.4 g/l. Her renal, liver and hemostasis function tests were normal. Chest imaging and cardiac ultrasounds were within normal limits. However, abdomino-pelvic imaging (ultrasound and scan) revealed the presence of a homogenous hepatosplenomegaly. Initially differential diagnosis mainly included brucellosis, typhoid fever, visceral leishmaniasis and hematological malignancies. Bacteriological cultures of blood (3 series), stool and urine revealed all negative. Hepatitis B Virus, *Salmonella* and *Brucella* antibody tests were also unrevealing. However, *Leishmania* rk39 immuno-chromatography test was positive, consistent with a VL diagnosis. A bone marrow aspiration was performed. Its parasitological examination revealed the presence of *Leishmania* amastigote forms. The patient serum was also positive for *Leishmania* anti-bodies by immunofluorescence antibody test (titer = 1/400). Blood parasite load determined by a real time quantitative PCR (qPCR) targeting the *Leishmania* kinetoplast was 400 parasites/ml [5].

**Figure 2:**
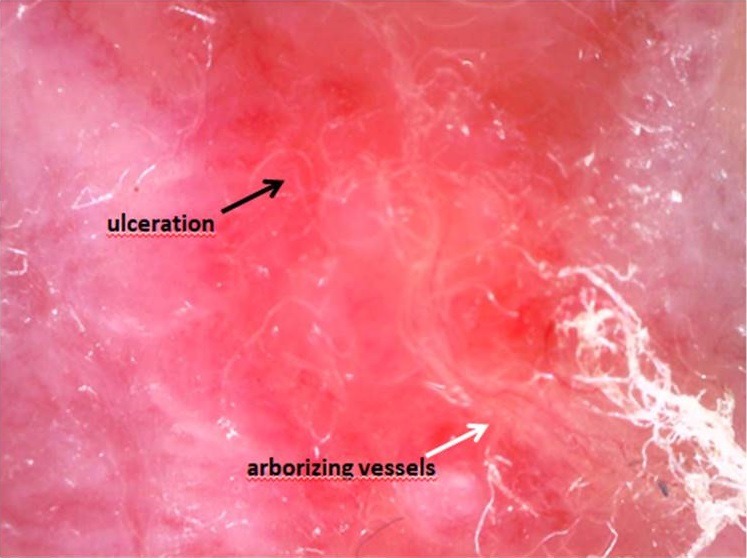
Ulcerations and short, fine arborizing vessels on dermoscopy

On another hand, direct microscopic examination performed on skin smears showed no *Leishmania* amastigotes. However, *Leishmania* DNA amplification was obtained from the dermal DNA extract, using the same qPCR. Molecular typing of isolates obtained from both samples (blood and skin) was carried out by sequence analysis of ribosomal internal transcribed spacer 1 (ITS1) region of rRNA gene. This analysis showed that the *Leishmania* strains from the arm cutaneous lesion and the blood corresponded to the species *L. infantum* and were 100% identical. Visceral Leishmaniasis diagnosis was retained and a course of anti-leishmanial therapy with intravenous amphotericin B deoxycholate at a dose of 1 g/kg/day every 2 days was started. The patient developed a mild renal failure, which was corrected after spacing the cures and rehydration. The response to treatment was rapidly favorable with apyrexia and an improvement in general conditions including resolution of fatigue and increased appetite. The splenomegaly resolved and blood count levels returned to reference ranges. Real-time PCR in blood after 28 days of treatment revealed negative. However, the skin lesion did not improve although *Leishmania* qPCR on dermal sample became negative. The physician reconsidered CL diagnosis and recommended a skin biopsy which was refused by the patient. A year later, the lesion was clinically almost the same (Figure 1 B). A polarized dermoscopy examination revealed ulcerations and short, fine arborizing vessels (Figure 2), evocative of a basal cell carcinoma. Histopathology examination of the skin lesion biopsy, finally accepted by the patient, confirmed the diagnosis of basal cell carcinoma. A complete excision of the tumor was performed and the evolution was good.

## Discussion

In classical VL, skin lesions are rare and patients usually do not recall any insect bite or lesion on inoculations' sites [1,3]. The concomitance of cutaneous and visceral syndromes is seen in 2 to 12% of HIV positive cases and mainly as a cutaneous co-morbidity with Kaposi's sarcoma [6]. However this phenomenon is rare among immunocompetent patients as it is reported in this observation [7]. In VL-HIV confection, skin lesions appear as symmetrical hypopigmented macules on the acral surfaces of the extremities or as violacious scaly plaques on the face [6]. These aspects were not observed in our patient (Figure 1 A). Another cutaneous syndrome associated with VL is Post-kala-azar dermal leishmaniasis (PKDL) mainly due to L. donovani in the Indian subcontinent [1]. Skin lesions are diffuse and typically develop after VL therapy, which was also not the case of our patient whose lesions preceded 2 or 3 years VL symptoms' onset. In 1992 Ayadi *et al*. reported a Tunisian case of unusual simultaneous skin manifestations (3 face-situated nodules) in a 19-month-old girl with VL [8]. However, the parasite was not evidenced in skin biopsies and *Leishmania* involvement was presumed only on the lesions' improvement after anti-leishmanial therapy. In our patient, DNA parasite detection confirmed the skin location of the parasite which highlights the better sensitivity of real-time PCR compared to smears microscopic examination [5,9].

Although VL is mainly a childhood disease in Tunisia, adult cases are not rare. Patients with no immunosuppressive factors, like our patient, are not uncommon and represent around 30% of adult VL cases [3]. Clinical symptoms during adult VL are frequently incomplete and less suggestive in comparison with those observed in infants [1,3]. However, for our patient, apart from the discreet hepatosplenomegaly, clinical and biological parameters were highly suggestive of the disease. The hypothesis that our patient' axilla cutaneous lesion corresponds to a primary CL infection is very unlikely. In fact, such site is not easily accessible to sandflies vectors and is rarely reported during CL surveys [2]. Moreover, the lesion did not heal under anti-leishmanial therapy and was subsequently confirmed as cancer. It corresponds probably to a secondarily colonization by *Leishmania* amastigotes after the parasite lymphatic or blood spread during the systemic VL infection. The cellular inflammatory environment locally induced by the basal cell carcinoma has certainly favored phagocytosis and local multiplication of the dispersed parasites which explain the positivity of specific qPCR [10]. In addition, ITS PCR-sequencing characterized strains from the 2 locations (blood and skin) as similar corresponding to L. infantum with 100% identity. The negativation of the skin *Leishmania* qPCR after specific treatment without any improvement of the lesion is a further proof that the cutaneous localization is secondary like a parasite metastasis in an auspicious inflammatory environment represented by the carcinoma [10].

## Conclusion

This report reveals that *Leishmania* spread during VL can induce secondary location of the parasite in cutaneous sites harboring inflammatory disturbances such as cancers.

## Competing interests

The authors declare no competing interests.
